# Olmesartan-Induced Spruelike Enteropathy: An Emerging Cause of Small Bowel Injury

**DOI:** 10.7759/cureus.9347

**Published:** 2020-07-22

**Authors:** Adnan Malik, Faisal Inayat, Muhammad Imran Malik, Muhammad Afzal, Muhammad F Azrak

**Affiliations:** 1 Hepatology, Loyola University Medical Center, Maywood, USA; 2 Internal Medicine, Allama Iqbal Medical College, Lahore, PAK; 3 Internal Medicine, Tameside and Glossop Integrated Care NHS Foundation Trust, Manchester, GBR; 4 Gastroenterology, William Beaumont Hospital, Royal Oak, USA

**Keywords:** olmesartan, diarrhea, spruelike enteropathy, adverse drug reaction, malabsorption

## Abstract

Olmesartan-induced spruelike enteropathy is a rare clinical entity that is characterized by unexplained chronic diarrhea and weight loss. Prompt recognition of this adverse event may be challenging due to clinical and histologic similarities with other small intestinal pathologies. We hereby delineate the case of an elderly female with a 14-month clinical history of non-bloody diarrhea and weight loss. After extensive diagnostic workup and exclusion of probable etiologies, the patient was diagnosed with olmesartan-associated enteropathy. A dramatic clinical and histologic recuperation was achieved after discontinuation of olmesartan. This paper illustrates the overarching need for a detailed clinical history focusing on medication review in patients presenting with chronic diarrhea with no obvious cause. The spruelike enteropathy associated with olmesartan is an emerging cause of small bowel injury. Clinicians should maintain a high index of suspicion for this adverse drug reaction. Early and correct diagnosis carries paramount importance in sparing these patients from unnecessary diagnostic investigations and therapeutic delays.

## Introduction

Rubio-Tapia et al. first described olmesartan-associated enteropathy in 2012 [1]. Since then, several case reports and small case series have been documented. While villous atrophy has also been reported with the use of other angiotensin II receptor blockers, olmesartan has been reported to have the highest incidence of such complications. Prior reports of similar side effects with telmisartan, irbesartan, valsartan, losartan, and eprosartan are also available [2]. This entity shares predominant symptoms and histologic characteristics with celiac disease. However, negative celiac serology, coupled with no response to a gluten-free diet, clearly denotes the distinct nature of this adverse drug reaction [1]. Even though the Food and Drug Administration (FDA) issued a warning for the risk of enteropathy, olmesartan remains a popular antihypertensive drug. It demonstrates a promising response in lowering blood pressure and bears a favorable drug-drug interaction profile [3]. In these patients, the symptoms of chronic diarrhea and weight loss are particularly disabling. These presentation patterns frequently prompt clinicians to conduct extensive diagnostic workup [4]. Therefore, this enteropathy not only impacts the quality of life in such patients but also leads to exhaustion of valuable hospital resources. Interestingly, the treatment of this abnormality is relatively simple, mainly consisting of discontinuation of olmesartan therapy [4,5]. The present paper adds to the existing clinical evidence pertaining to this severe adverse reaction of olmesartan and serves the purpose of community awareness for early etiology establishment in affected patients. This case study has previously been presented as an abstract (Abstract: Malik A, Alsabbak H, Samreen A, Siddique K, Ashraf H, Zakharia K, Azrak M. Drug-Induced Flu-Like Enteropathy. Annual Scientific Meeting, American College of Gastroenterology; October 05-10, 2018, Philadelphia, Pennsylvania).

## Case presentation

A 75-year-old Caucasian female presented to our medical center with frequent nocturnal, non-bloody, loose stools for the past 14 months. The diarrhea was also associated with nausea, vomiting, and weight loss (100 lbs). Conservative management resulted in only temporary relief. Esophagogastroduodenoscopy (EGD) was performed 13 months ago, which revealed hiatal hernia with chronic active gastritis. Her colonoscopy was normal. A trial of a gluten-free diet failed to resolve her symptoms. In addition, the patient’s medical history was significant for hypertension, hyperlipidemia, osteoarthritis, chronic obstructive pulmonary disease, hypothyroidism, and diabetes mellitus. At presentation, she had been adhering to antihypertensive therapy with olmesartan medoxomil (Benicar; Daiichi Sankyo, Inc., Basking Ridge, New Jersey) 40 mg/day over the past several years. Her other home medications included albuterol, budesonide, fluticasone, levothyroxine, insulin glargine, potassium fiber capsules, and vitamin B_12_. On admission, her blood pressure was 80/49 mm Hg, and heart rate was 100 beats per minute. Abdominal examination was unremarkable for abnormalities.

Investigations

**Figure 1:**
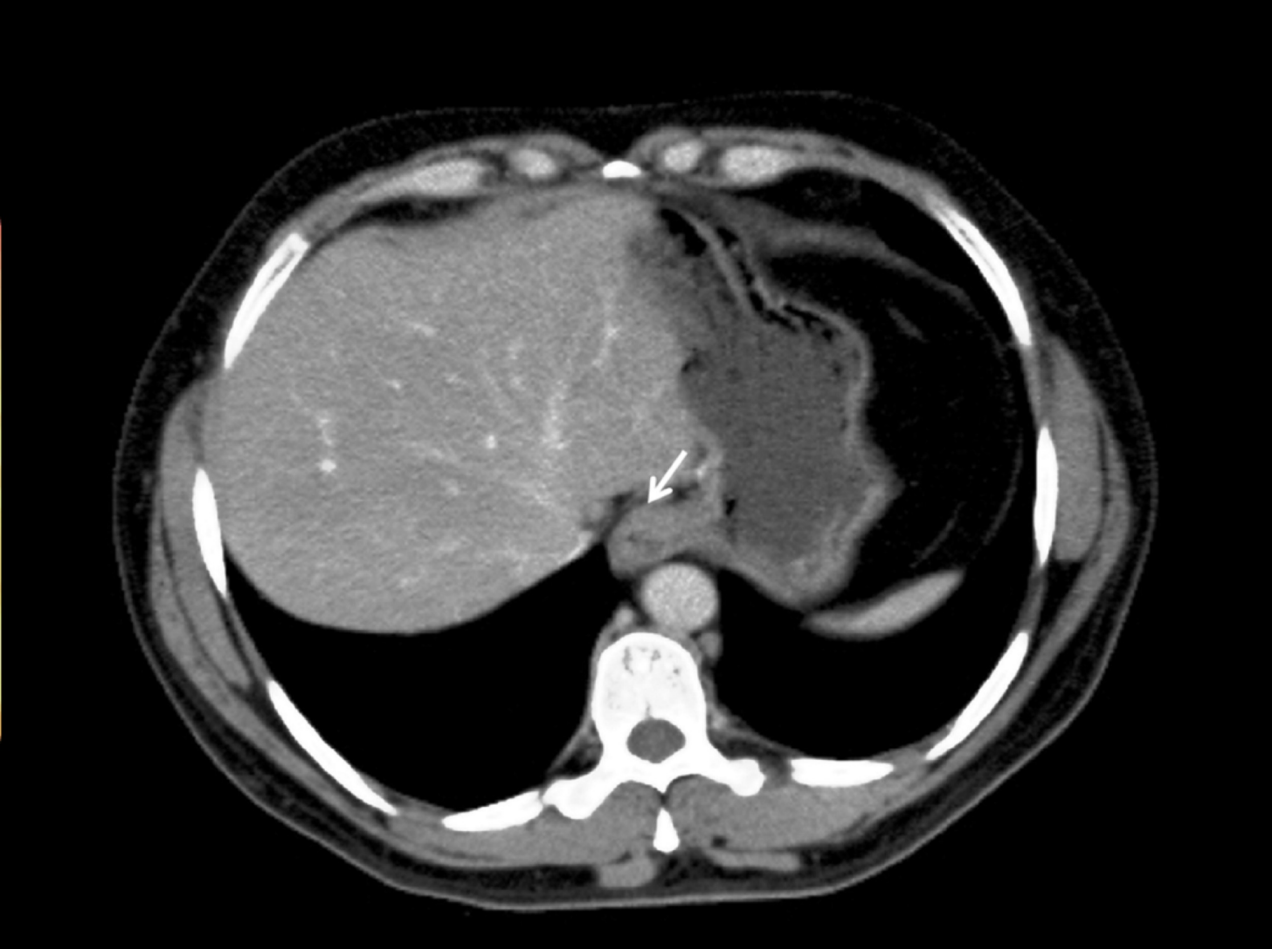
Contrast-enhanced computed tomography axial section (soft tissue window) at the level of diaphragm showing herniation of the part of the stomach through the esophageal hiatus into the thoracic cavity (arrow).

Laboratory evaluation revealed mild anemia, hyperchloremia (120 mEq/L), hypoalbuminemia (2.6 g/dL), and low serum bicarbonate levels (16 mEq/L). The findings of initial testing showed that the patient was in metabolic acidosis. Vitamin B_12_ 186 ng/mL (seven months ago), vasoactive intestinal polypeptide <13 pmol/L, and gastrin was <25 pg/mL. Her serum creatinine level was mildly elevated to 1.2 mg/dL (baseline: 1.1 mg/dL). Erythrocyte sedimentation rate (ESR), serum lipase and amylase, and liver function tests were within normal limits. Stool leukocyte and fecal occult blood tests were positive. Stool cultures for ova, parasites, and *Clostridium difficile *toxins were negative. Fecal fat testing, *Cryptosporidium *and *Giardia *antigens, celiac panel, and human immunodeficiency virus testing came out negative. Contrast-enhanced computed tomography showed herniation of the part of the stomach through the esophageal hiatus into the thoracic cavity, consistent with hiatal hernia (Figure 1).

**Figure 2:**
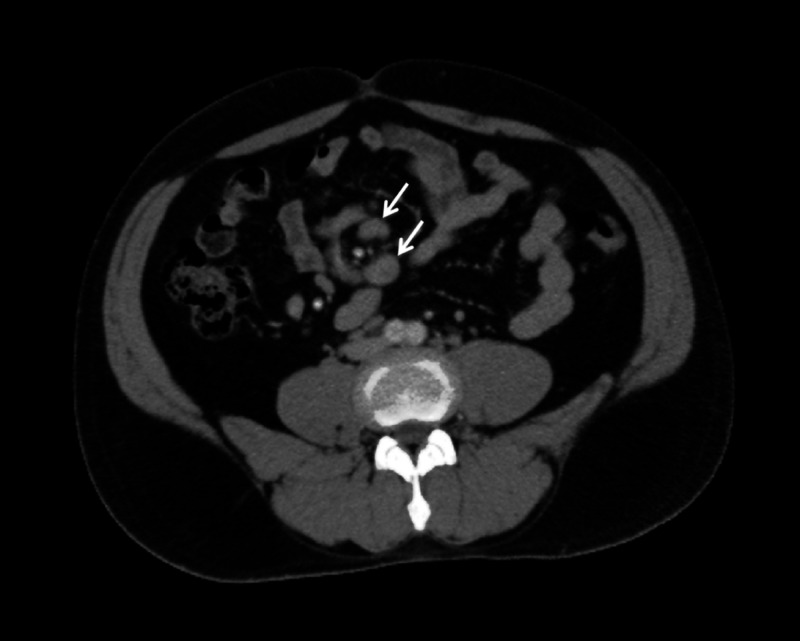
Computed tomography post-intravenous contrast axial section (soft tissue window) at the level of aortic bifurcation showing enlarged mesenteric lymph nodes (arrows).

Computed tomography scan further divulged enlarged mesenteric lymph nodes, measuring 9-12 mm in the short axis (Figure 2).

**Figure 3:**
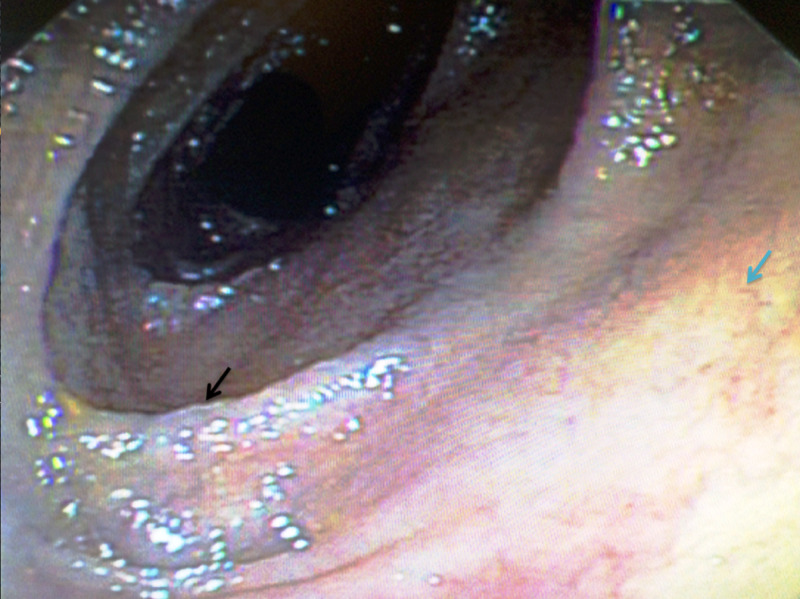
Upper endoscopy showing scalloped duodenal folds (black arrow) and mosaic mucosal pattern, consistent with cracked-mud appearance (blue arrow).

EGD showed scalloped duodenal folds and a mosaic mucosal pattern, consistent with cracked-mud appearance (Figure 3).

**Figure 4:**
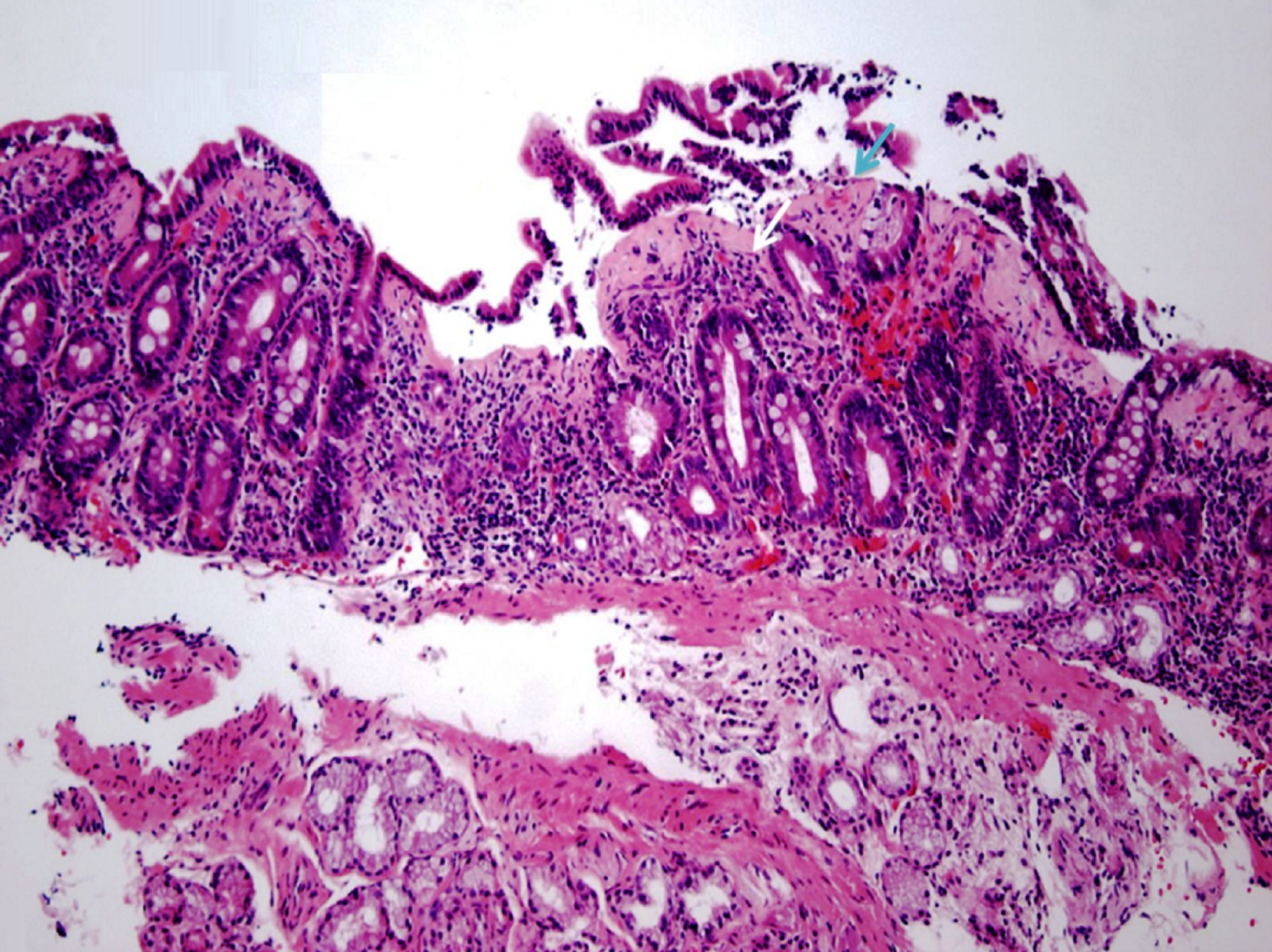
Pathologic examination of the duodenal biopsy specimen showing partial villous atrophy (blue arrow) with associated increased intraepithelial lymphocytes (white arrow). (Hematoxylin and eosin staining; 100x)

Minimal endoscopic changes were also noted in the colon and terminal ileum. Pathologic examination of the biopsy specimens from the duodenum showed chronic partial villous atrophy with increased intraepithelial lymphocytes (Figure 4).

**Figure 5:**
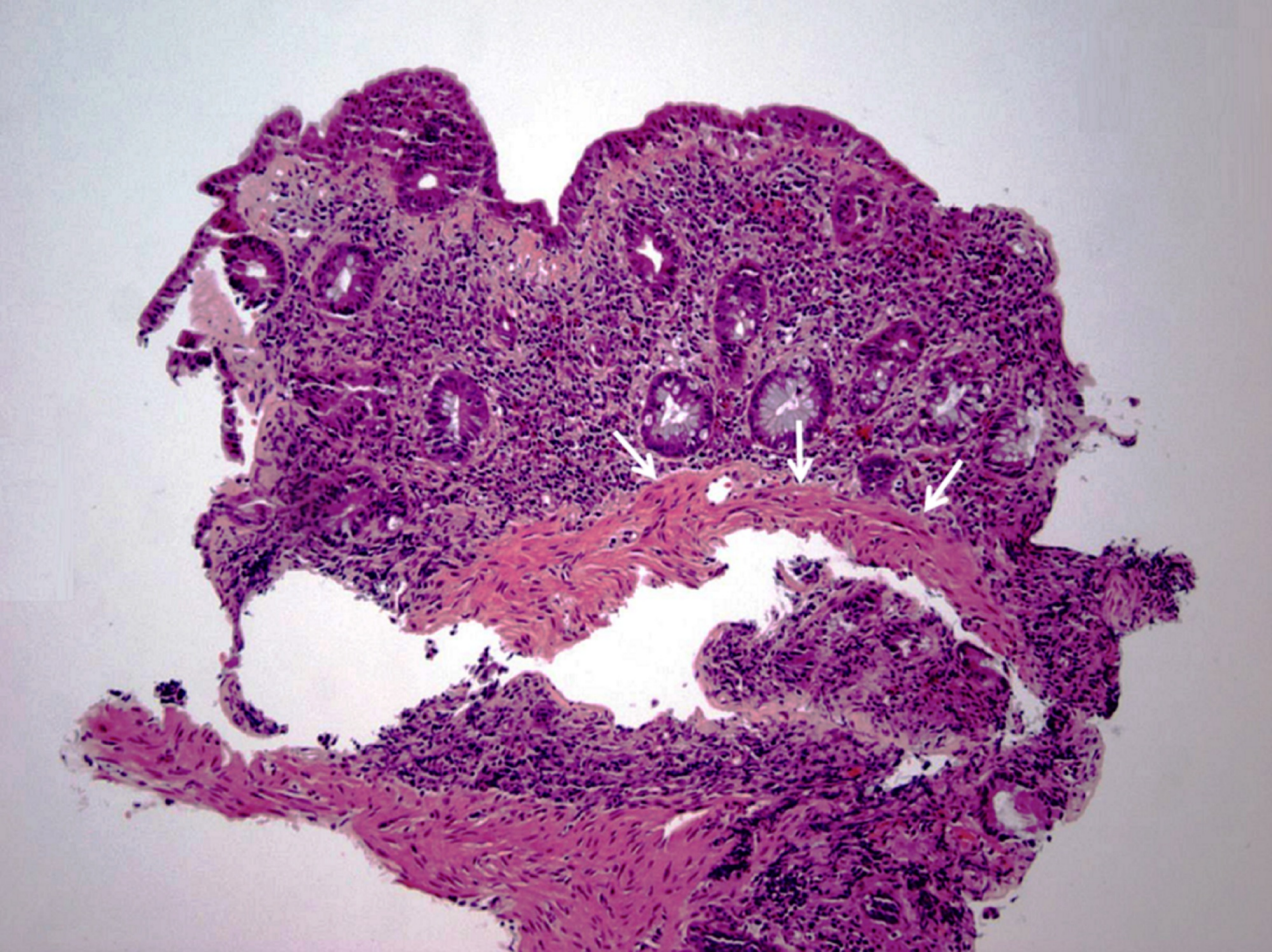
Duodenal biopsy showing a thickened subepithelial collagen band (arrows). (Hematoxylin and eosin staining; 100x)

A thickened subepithelial collagen table was another prominent histologic finding (Figure 5).

Increased gastric submucosal lymphocytes and collagen deposits were also noted. Furthermore, chronic colitis with focal collagenous deposits was evident but random colonic biopsies ruled out microscopic colitis.

Differential diagnosis

The differentials included drug-induced and autoimmune enteropathies, hypogammaglobulinemia-associated sprue, tropical sprue, small intestinal bacterial overgrowth, Giardiasis, microscopic colitis, intestinal lymphoma, *Clostridium difficile* colitis, Whipple's disease, collagenous sprue or unclassified sprue. On the basis of workup findings and exclusion of probable etiologies, she was eventually diagnosed with olmesartan-induced spruelike enteropathy.

Treatment

The patient was educated about her disease and was directed to immediately discontinue olmesartan. She was managed conservatively for gastrointestinal complaints and was prescribed alternate medication for hypertension.

Outcome and follow-up

**Figure 6:**
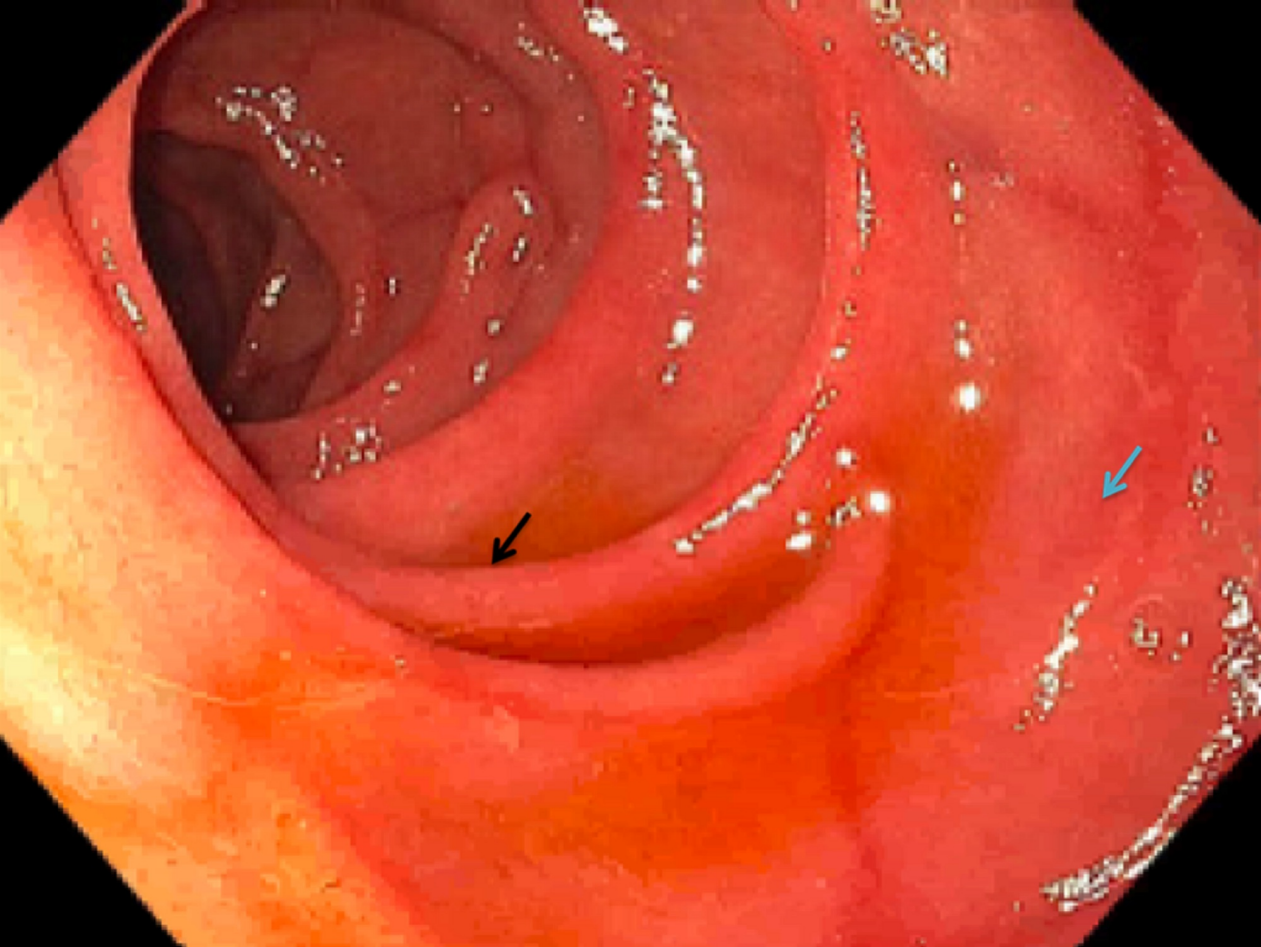
Repeat upper endoscopy performed after six months showing normal duodenal folds (black arrow) and macroscopically normal mucosa (blue arrow).

After six weeks of discontinuing olmesartan, she showed clinical remission of her symptoms and signs. She began thriving, as reflected by her weight gain (5 lbs). The complete clinical resolution of her gastrointestinal symptoms was achieved in three months. Repeat EGD performed after six months showed macroscopically normal duodenal folds and mucosa (Figure 6).

**Figure 7:**
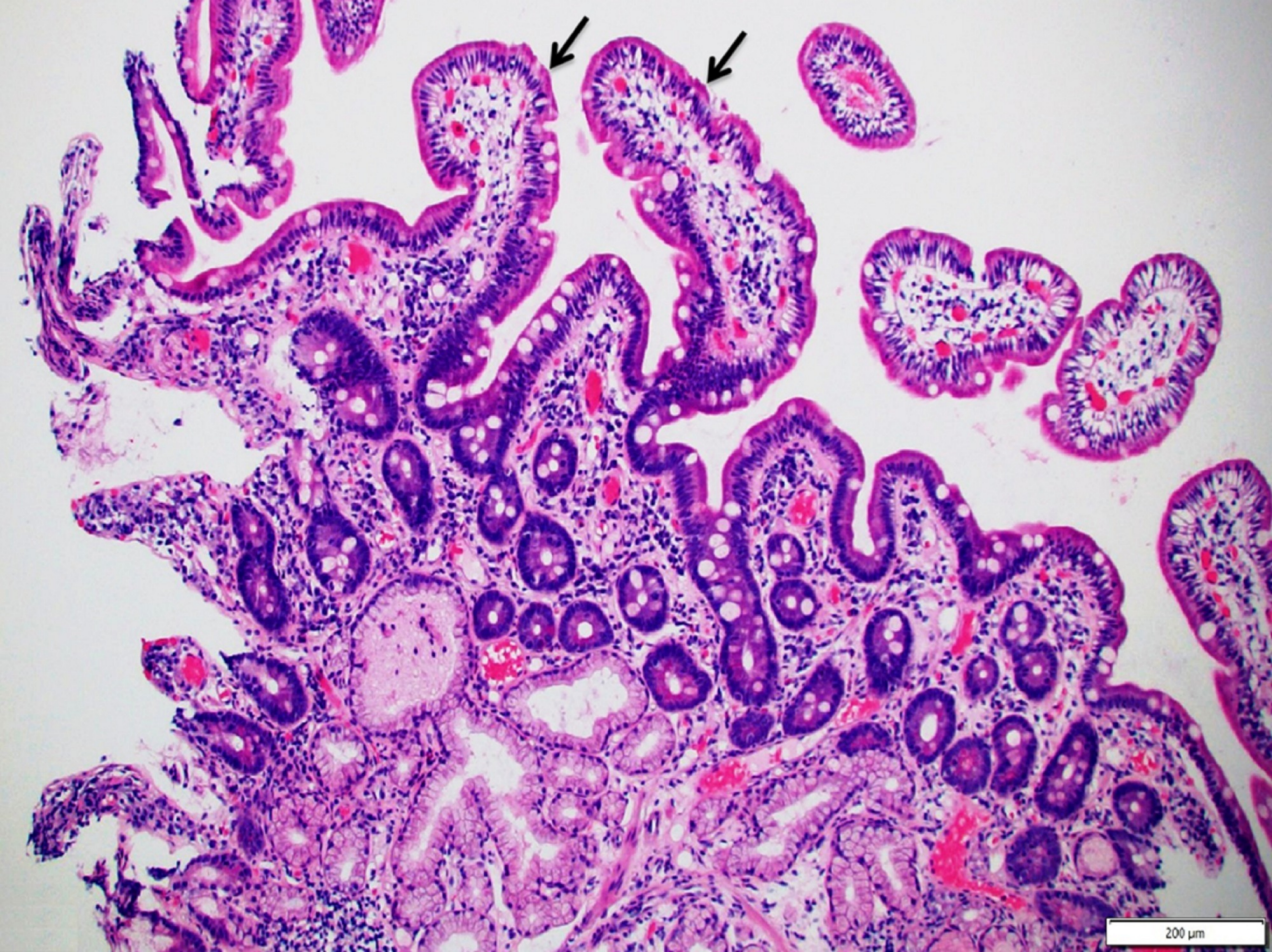
Repeat duodenal biopsy showing normal duodenal villous architecture (arrows). (Hematoxylin and eosin staining; 100x)

Pathologic examination of the biopsy specimens also showed histological recovery (Figure 7).

The patient has been symptom-free and continues to do well to date.

## Discussion

Olmesartan is a widely used angiotensin II receptor blocker for hypertension. According to one estimate, around 45.3 million prescriptions were dispensed in the United States through the year 2011 [6]. The overall tolerability profile of this drug has been good, with only a few minor side effects such as dizziness, flu-like symptoms, or headache [7]. However, clinicians at Mayo Clinic identified a novel entity of severe spruelike enteropathy secondary to the use of olmesartan in 2012 [1]. Several case reports and small case series of this adverse drug reaction have been described thus far. Published medical literature demonstrates that this reaction shows no clear gender predominance. It mostly involves patients in their seventh and eighth decades of life [8]. The clinical presentation of olmesartan-related spruelike enteropathy is routinely characterized by chronic non-bloody diarrhea, vomiting, crampy abdominal pain, and weight loss [9]. Occasionally, the aforementioned symptoms may lead to dehydration, electrolyte imbalance, or acute kidney injury [10]. While it remains extremely rare, gastrointestinal perforation can also occur in such patients [11].

The exact pathogenesis of this adverse drug reaction is unknown. However, severe intestinal inflammation and mucosal damage following the chronic use of olmesartan implicate cell-mediated immune response [12]. In a majority of patients with severe disease, the presence of either HLA-DQ2 or DQ8 haplotypes has been identified. This observation further endorses the altered immune response as the likely causal mechanism [12]. Furthermore, increased levels of transforming growth factor-beta (TGF-β) have been considered as causative factors for intestinal epithelial injury. The elevated levels of TGF-β have been thought to be triggered by the increased levels of angiotensin II in such patients [12]. The delay between the initiation of olmesartan treatment and disease activity also supports immune-mediated pathophysiology. The length of the time period between olmesartan exposure and symptom-onset is variable, ranging from a few months to several years. Based on existing clinical data, the mean duration of this time interval has been 3.1 years [9].

Laboratory evaluation predominantly reveals a severe malabsorption process. Anemia, hypoalbuminemia, electrolyte imbalance, and vitamin deficiencies develop after long-term use of olmesartan. Given the overlapping clinical and histological patterns with celiac disease, negative celiac serology is imperative in the detection of olmesartan-associated disease [13]. The endoscopic appearance of this lesion is mostly non-specific. Nodular mucosal changes in the duodenum with partial or complete villous atrophy and ulcerations can be found [13]. It is notable that concurrent involvement of the entire gastrointestinal tract has also been observed. Endoscopic biopsy is an important diagnostic investigation. Pathologic examination of biopsy specimens commonly shows total or partial duodenal villous atrophy, mucosal granulocytic infiltration, and a thickened subepithelial collagen layer [9,12,14]. Conversely, a remarkable histologic variation may exist in these patients. In that context, endoscopic biopsy findings alone cannot be considered as reliable diagnostic markers due to the lack of statistically significant data [15]. Olmesartan cessation results in prompt clinical improvement in most patients. Therefore, an olmesartan-free interval can also help in the identification of this etiology in difficult-to-diagnose cases. It is notable that the definitive diagnosis can only be made after the resolution of clinical and histological findings following olmesartan cessation.

With regard to treatment, immediate olmesartan discontinuation is imperative. The median time to resolution of individual symptoms varies from case to case. It may require a few weeks to several months in order to achieve symptomatic relief [16]. The small bowel mucosal changes mostly disappear in three to six months of the drug withdrawal. Therefore, a follow-up endoscopic biopsy is warranted to gauge the mucosal improvement. Notably, some patients may only show a partial response to olmesartan cessation alone [17]. In such cases, additional treatment with budesonide may be employed. The monitoring of the progression of severe and recurrent olmesartan-induced spruelike enteropathy is important after the cessation of the implicated drug [17].

The patient involved in this study demonstrated clinical symptoms related to celiac disease. However, no response to a gluten-free diet and negative serology provided a vital diagnostic clue that precluded a diagnosis of celiac disease. The endoscopic biopsy was remarkable for partial villous atrophy, suggesting a possible drug-induced injury. After a careful review of the medications, olmesartan was considered the culprit drug. It was immediately discontinued and her symptoms showed improvement. Chronic diarrhea and weight loss were particularly disabling in this patient. Therefore, if the diagnosis had been made early in the course of the disease, she could have been spared from these significant problems. Furthermore, extensive diagnostic workup was performed, which turned out to be negative for all probable etiologies. It not only delayed the diagnosis but also exhausted valuable hospital resources. Thus, physicians should remain vigilant and include olmesartan-induced spruelike enteropathy in the differentials of chronic diarrhea to avoid such predicaments.

## Conclusions

This article highlights the pathologic association between olmesartan and spruelike enteropathy. This adverse drug reaction may closely mimic several intestinal disorders warranting extreme clinical vigilance in such patients. Although it is a relatively rare clinical entity, physicians should be aware of this adverse event to avoid extensive workup and treatment delays. Prospective studies evaluating the serological and histological profiles of olmesartan-induced spruelike enteropathy are required to further elucidate the associated diagnostic and therapeutic conundrums.
